# Bis[4-(dimethyl­amino)pyridinium] 3.75-bromido-0.25-chloridodiphenyl­plumbate(IV)

**DOI:** 10.1107/S1600536808040312

**Published:** 2008-12-06

**Authors:** Kong Mun Lo, Seik Weng Ng

**Affiliations:** a Department of Chemistry, University of Malaya, 50603 Kuala Lumpur, Malaysia

## Abstract

The Pb^IV^ atom of the plumbate dianion in the title compound, (C_7_H_11_N)_2_[Pb(Br_3.75_Cl_0.25_)(C_6_H_5_)_2_], lies on a centre of inversion in a tetra­gonally compressed octa­hedral geometry. One of the attached Br atoms is disordered with respect to a Cl atom in a 7:1 ratio. The disordered halogen atom is an N—H⋯(Br/Cl) hydrogen-bond acceptor for the cation.

## Related literature

For the structure of the isostructural compound bis­(4-di­methyl­amino­pyridinium) tetra­bromidodiphenyl­plumbate(IV), see: Lo & Ng (2008 ▶).
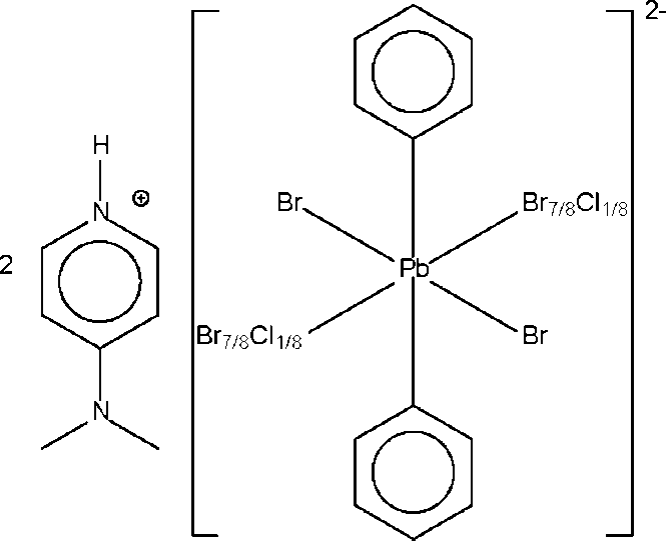


## Experimental

### 

#### Crystal data

(C_7_H_11_N)_2_[Pb(Br_3.75_Cl_0.25_)(C_6_H_5_)_2_]
*M* *_r_* = 916.27
Monoclinic, 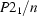

*a* = 9.5010 (2) Å
*b* = 13.8916 (3) Å
*c* = 10.9851 (2) Å
β = 92.996 (1)°
*V* = 1447.88 (5) Å^3^
*Z* = 2
Mo *K*α radiation
μ = 11.05 mm^−1^
*T* = 100 (2) K
0.12 × 0.11 × 0.10 mm

#### Data collection

Bruker SMART APEX CCD diffractometer
Absorption correction: multi-scan (*SADABS*; Sheldrick, 1996 ▶) *T*_min_ = 0.351, *T*_max_ = 0.405 (expected range = 0.287–0.331)
10028 measured reflections
3327 independent reflections
2909 reflections with *I* > 2σ(*I*)
*R*_int_ = 0.028

#### Refinement

*R*[*F* ^2^ > 2σ(*F* ^2^)] = 0.022
*wR*(*F* ^2^) = 0.048
*S* = 1.02
3327 reflections
166 parameters
1 restraint
H atoms treated by a mixture of independent and constrained refinement
Δρ_max_ = 0.77 e Å^−3^
Δρ_min_ = −0.52 e Å^−3^

### 

Data collection: *APEX2* (Bruker, 2007 ▶); cell refinement: *SAINT* (Bruker, 2007 ▶); data reduction: *SAINT*; program(s) used to solve structure: *SHELXS97* (Sheldrick, 2008 ▶); program(s) used to refine structure: *SHELXL97* (Sheldrick, 2008 ▶); molecular graphics: *X-SEED* (Barbour, 2001 ▶); software used to prepare material for publication: *publCIF* (Westrip, 2008 ▶).

## Supplementary Material

Crystal structure: contains datablocks global, I. DOI: 10.1107/S1600536808040312/hb2863sup1.cif

Structure factors: contains datablocks I. DOI: 10.1107/S1600536808040312/hb2863Isup2.hkl

Additional supplementary materials:  crystallographic information; 3D view; checkCIF report

## Figures and Tables

**Table 1:** Selected bond lengths (Å) *X* = Br, Cl.

Pb1—C1	2.184 (3)
Pb1—*X*1	2.8523 (3)
Pb1—Br2	2.8885 (3)

**Table 2:** Hydrogen-bond geometry (Å, °) *X* = Br, Cl.

*D*—H⋯*A*	*D*—H	H⋯*A*	*D*⋯*A*	*D*—H⋯*A*
N1—H1⋯*X*1	0.87 (1)	2.49 (2)	3.260 (3)	148 (4)

**Fig. 1.**
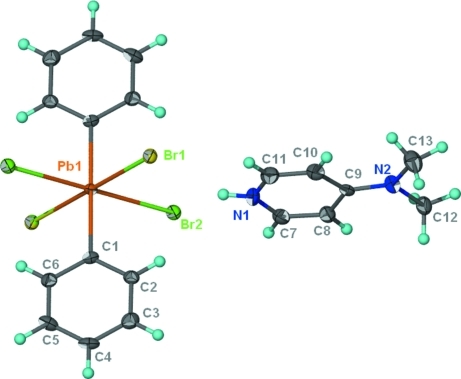
View of the molecular structure of (I) at the 70% probability level. H atoms are drawn as spheres of arbitrary radius. Unlabelled atoms in the anion are generated by the symmetry operation (1 - x, 1 - y, 1 - z).

## supplementary crystallographic information

### Experimental

Diphenyllead dichloride (1.3 g, 3 mmol) and 4-dimethylaminopyridine hydrobromide perbromide (1.1 g, 3 mmol) were heated in chloroform (100 ml) for an hour. The filtered solution when allowed to evaporate yielded large colorless crystals of (I).

### Refinement

The carbon-bound H-atoms were placed in calculated positions (C—H = 0.95–0.98 Å) and refined as riding with *U*_iso_(H) = 1.2 to 1.5*U*_eq_(C). The ammonium H atom was located in a difference Fourier map, and was refined with a distance constraint of N—H = 0.88 (1) Å; its U_iso_ value was refined.

The two independent halogen atoms were initially refined as full-occupancy Br atoms; however, the difference Fourier map had a deep hole near one of the two. When this atom was allowed to refine as a mixture of bromine and chlorine, the refinement converged, and it gave the Br:Cl ratio as 0.88:0.12. The ratio was subsequently fixed as 0.875:0.125. Attempts to model the Br and Cl atoms on separate sites were not successful.

The published (C_7_H_11_N)_2_[PbBr_4_(C_6_H_5_)_2_] structure (Lo & Ng, 2008) does not contain any chlorine as the compound was synthesized by the cleavage of tetraphenyllead by 4-aminomethylpyridine hydrobromide perbromide.

### Crystal data

**Table:**

(C_7_H_11_N)_2_[PbBr_3.75_(C_6_H_5_)_2_Cl_0.25_]	*F*(000) = 867
*M**_r_* = 916.27	*D*_x_ = 2.102 Mg m^−^^3^
Monoclinic, *P*2_1_/*n*	Mo *K*α radiation, λ = 0.71073 Å
Hall symbol: -P 2yn	Cell parameters from 4255 reflections
*a* = 9.5010 (2) Å	θ = 2.4–28.4°
*b* = 13.8916 (3) Å	µ = 11.05 mm^−^^1^
*c* = 10.9851 (2) Å	*T* = 100 K
β = 92.996 (1)°	Faceted block, colourless
*V* = 1447.88 (5) Å^3^	0.12 × 0.11 × 0.10 mm
*Z* = 2	

### Data collection

**Table:**

Bruker SMART APEX CCD diffractometer	3327 independent reflections
Radiation source: fine-focus sealed tube	2909 reflections with *I* > 2σ(*I*)
graphite	*R*_int_ = 0.028
ω scans	θ_max_ = 27.5°, θ_min_ = 2.4°
Absorption correction: multi-scan (*SADABS*; Sheldrick, 1996)	*h* = −12→12
*T*_min_ = 0.351, *T*_max_ = 0.405	*k* = −15→18
10028 measured reflections	*l* = −14→14

### Refinement

**Table:**

Refinement on *F*^2^	Primary atom site location: structure-invariant direct methods
Least-squares matrix: full	Secondary atom site location: difference Fourier map
*R*[*F*^2^ > 2σ(*F*^2^)] = 0.022	Hydrogen site location: inferred from neighbouring sites
*wR*(*F*^2^) = 0.048	H atoms treated by a mixture of independent and constrained refinement
*S* = 1.02	*w* = 1/[σ^2^(*F*_o_^2^) + (0.0227*P*)^2^ + 0.2619*P*] where *P* = (*F*_o_^2^ + 2*F*_c_^2^)/3
3327 reflections	(Δ/σ)_max_ = 0.001
166 parameters	Δρ_max_ = 0.77 e Å^−^^3^
1 restraint	Δρ_min_ = −0.52 e Å^−^^3^

### Special details

**Table:**

Geometry. All e.s.d.'s (except the e.s.d. in the dihedral angle between two l.s. planes) are estimated using the full covariance matrix. The cell e.s.d.'s are taken into account individually in the estimation of e.s.d.'s in distances, angles and torsion angles; correlations between e.s.d.'s in cell parameters are only used when they are defined by crystal symmetry. An approximate (isotropic) treatment of cell e.s.d.'s is used for estimating e.s.d.'s involving l.s. planes.

### Fractional atomic coordinates and isotropic or equivalent isotropic displacement parameters (Å^2^)

**Table:**

	*x*	*y*	*z*	*U*_iso_*/*U*_eq_	Occ. (<1)
Pb1	0.5000	0.5000	0.5000	0.01182 (5)	
Br1	0.55819 (4)	0.61268 (2)	0.29147 (3)	0.01476 (8)	0.875
Br2	0.76221 (3)	0.40251 (2)	0.46037 (3)	0.01660 (8)	
Cl1	0.55819 (4)	0.61268 (2)	0.29147 (3)	0.01476 (8)	0.125
N1	0.8894 (3)	0.5871 (2)	0.2345 (3)	0.0224 (7)	
H1	0.815 (3)	0.582 (3)	0.277 (3)	0.042 (13)*	
N2	1.1998 (3)	0.59111 (19)	−0.0113 (2)	0.0173 (6)	
C1	0.6143 (3)	0.6021 (2)	0.6197 (3)	0.0138 (6)	
C2	0.7445 (3)	0.6367 (2)	0.5890 (3)	0.0152 (7)	
H2	0.7856	0.6156	0.5167	0.018*	
C3	0.8144 (4)	0.7030 (2)	0.6660 (3)	0.0198 (7)	
H3	0.9040	0.7271	0.6465	0.024*	
C4	0.7533 (4)	0.7336 (2)	0.7709 (3)	0.0191 (7)	
H4	0.8009	0.7790	0.8229	0.023*	
C5	0.6228 (4)	0.6982 (2)	0.8003 (3)	0.0204 (7)	
H5	0.5814	0.7194	0.8724	0.024*	
C6	0.5527 (4)	0.6321 (2)	0.7248 (3)	0.0163 (7)	
H6	0.4635	0.6076	0.7448	0.020*	
C7	1.0060 (4)	0.5338 (3)	0.2555 (3)	0.0208 (7)	
H7	1.0141	0.4941	0.3260	0.025*	
C8	1.1124 (4)	0.5356 (2)	0.1781 (3)	0.0189 (7)	
H8	1.1945	0.4980	0.1953	0.023*	
C9	1.1019 (3)	0.5933 (2)	0.0715 (3)	0.0146 (7)	
C10	0.9798 (4)	0.6523 (2)	0.0576 (3)	0.0189 (7)	
H10	0.9701	0.6959	−0.0089	0.023*	
C11	0.8778 (4)	0.6469 (2)	0.1382 (3)	0.0220 (8)	
H11	0.7962	0.6860	0.1267	0.026*	
C12	1.3282 (4)	0.5343 (3)	0.0091 (3)	0.0225 (7)	
H12A	1.3034	0.4683	0.0313	0.034*	
H12B	1.3872	0.5632	0.0752	0.034*	
H12C	1.3803	0.5332	−0.0656	0.034*	
C13	1.1938 (4)	0.6535 (3)	−0.1175 (3)	0.0267 (8)	
H13A	1.0952	0.6658	−0.1434	0.040*	
H13B	1.2414	0.6222	−0.1840	0.040*	
H13C	1.2409	0.7146	−0.0970	0.040*	

### Atomic displacement parameters (Å^2^)

**Table:**

	*U*^11^	*U*^22^	*U*^33^	*U*^12^	*U*^13^	*U*^23^
Pb1	0.01154 (9)	0.01443 (9)	0.00939 (8)	−0.00187 (6)	−0.00034 (6)	−0.00126 (6)
Br1	0.01359 (18)	0.01750 (17)	0.01320 (16)	0.00047 (13)	0.00073 (13)	0.00243 (12)
Br2	0.01506 (17)	0.02009 (17)	0.01462 (16)	0.00203 (12)	0.00038 (12)	−0.00043 (12)
Cl1	0.01359 (18)	0.01750 (17)	0.01320 (16)	0.00047 (13)	0.00073 (13)	0.00243 (12)
N1	0.0161 (17)	0.0309 (17)	0.0206 (16)	−0.0016 (13)	0.0056 (12)	−0.0050 (13)
N2	0.0201 (16)	0.0158 (14)	0.0162 (14)	0.0043 (11)	0.0029 (11)	0.0024 (11)
C1	0.0133 (17)	0.0130 (16)	0.0146 (16)	−0.0008 (12)	−0.0031 (12)	0.0003 (12)
C2	0.0157 (17)	0.0171 (16)	0.0127 (16)	0.0016 (13)	−0.0020 (12)	0.0004 (12)
C3	0.0186 (19)	0.0201 (18)	0.0204 (18)	−0.0036 (14)	−0.0008 (14)	0.0032 (13)
C4	0.024 (2)	0.0171 (18)	0.0156 (17)	−0.0024 (14)	−0.0081 (14)	−0.0015 (13)
C5	0.029 (2)	0.0191 (18)	0.0133 (16)	−0.0003 (14)	−0.0015 (14)	−0.0027 (13)
C6	0.0147 (18)	0.0197 (17)	0.0143 (16)	0.0007 (13)	−0.0017 (13)	0.0031 (13)
C7	0.022 (2)	0.0252 (18)	0.0149 (17)	−0.0029 (15)	0.0004 (14)	−0.0010 (14)
C8	0.0188 (18)	0.0211 (17)	0.0170 (17)	0.0021 (14)	0.0014 (13)	−0.0004 (13)
C9	0.0158 (17)	0.0164 (16)	0.0113 (15)	−0.0013 (12)	−0.0011 (12)	−0.0051 (12)
C10	0.0213 (19)	0.0171 (17)	0.0183 (18)	0.0024 (14)	−0.0009 (14)	−0.0008 (13)
C11	0.0188 (19)	0.0229 (19)	0.0242 (19)	0.0047 (14)	−0.0015 (14)	−0.0052 (14)
C12	0.0190 (19)	0.0306 (19)	0.0180 (18)	0.0059 (15)	0.0027 (14)	0.0008 (15)
C13	0.032 (2)	0.028 (2)	0.0203 (19)	0.0065 (16)	0.0057 (16)	0.0057 (15)

### Geometric parameters (Å, °)

**Table:**

Pb1—C1^i^	2.184 (3)	C4—C5	1.388 (5)
Pb1—C1	2.184 (3)	C4—H4	0.9500
Pb1—Br1	2.8523 (3)	C5—C6	1.385 (4)
Pb1—Cl1^i^	2.8523 (3)	C5—H5	0.9500
Pb1—Br1^i^	2.8523 (3)	C6—H6	0.9500
Pb1—Br2	2.8885 (3)	C7—C8	1.355 (4)
Pb1—Br2^i^	2.8885 (3)	C7—H7	0.9500
N1—C7	1.342 (5)	C8—C9	1.419 (4)
N1—C11	1.345 (5)	C8—H8	0.9500
N1—H1	0.87 (1)	C9—C10	1.421 (4)
N2—C9	1.335 (4)	C10—C11	1.348 (5)
N2—C13	1.452 (4)	C10—H10	0.9500
N2—C12	1.461 (4)	C11—H11	0.9500
C1—C6	1.385 (4)	C12—H12A	0.9800
C1—C2	1.385 (4)	C12—H12B	0.9800
C2—C3	1.395 (4)	C12—H12C	0.9800
C2—H2	0.9500	C13—H13A	0.9800
C3—C4	1.383 (4)	C13—H13B	0.9800
C3—H3	0.9500	C13—H13C	0.9800
			
C1^i^—Pb1—C1	180.00 (12)	C3—C4—C5	120.2 (3)
C1^i^—Pb1—Br1	89.09 (8)	C3—C4—H4	119.9
C1—Pb1—Br1	90.91 (8)	C5—C4—H4	119.9
C1^i^—Pb1—Cl1^i^	90.91 (8)	C6—C5—C4	120.2 (3)
C1—Pb1—Cl1^i^	89.09 (8)	C6—C5—H5	119.9
Br1—Pb1—Cl1^i^	180.0	C4—C5—H5	119.9
C1^i^—Pb1—Br1^i^	90.91 (8)	C5—C6—C1	119.2 (3)
C1—Pb1—Br1^i^	89.09 (8)	C5—C6—H6	120.4
Br1—Pb1—Br1^i^	180.0	C1—C6—H6	120.4
Cl1^i^—Pb1—Br1^i^	0.000 (17)	N1—C7—C8	121.2 (3)
C1^i^—Pb1—Br2	90.60 (8)	N1—C7—H7	119.4
C1—Pb1—Br2	89.40 (8)	C8—C7—H7	119.4
Br1—Pb1—Br2	86.065 (9)	C7—C8—C9	120.4 (3)
Cl1^i^—Pb1—Br2	93.935 (9)	C7—C8—H8	119.8
Br1^i^—Pb1—Br2	93.935 (9)	C9—C8—H8	119.8
C1^i^—Pb1—Br2^i^	89.40 (8)	N2—C9—C8	121.9 (3)
C1—Pb1—Br2^i^	90.60 (8)	N2—C9—C10	122.3 (3)
Br1—Pb1—Br2^i^	93.935 (9)	C8—C9—C10	115.9 (3)
Cl1^i^—Pb1—Br2^i^	86.065 (9)	C11—C10—C9	120.5 (3)
Br1^i^—Pb1—Br2^i^	86.065 (9)	C11—C10—H10	119.7
Br2—Pb1—Br2^i^	180.0	C9—C10—H10	119.7
C7—N1—C11	120.6 (3)	N1—C11—C10	121.2 (3)
C7—N1—H1	124 (3)	N1—C11—H11	119.4
C11—N1—H1	116 (3)	C10—C11—H11	119.4
C9—N2—C13	122.3 (3)	N2—C12—H12A	109.5
C9—N2—C12	120.9 (3)	N2—C12—H12B	109.5
C13—N2—C12	116.3 (3)	H12A—C12—H12B	109.5
C6—C1—C2	121.3 (3)	N2—C12—H12C	109.5
C6—C1—Pb1	118.6 (2)	H12A—C12—H12C	109.5
C2—C1—Pb1	120.1 (2)	H12B—C12—H12C	109.5
C1—C2—C3	119.0 (3)	N2—C13—H13A	109.5
C1—C2—H2	120.5	N2—C13—H13B	109.5
C3—C2—H2	120.5	H13A—C13—H13B	109.5
C4—C3—C2	120.1 (3)	N2—C13—H13C	109.5
C4—C3—H3	120.0	H13A—C13—H13C	109.5
C2—C3—H3	120.0	H13B—C13—H13C	109.5
			
Br1—Pb1—C1—C6	133.5 (2)	C4—C5—C6—C1	−0.2 (5)
Cl1^i^—Pb1—C1—C6	−46.5 (2)	C2—C1—C6—C5	0.2 (5)
Br1^i^—Pb1—C1—C6	−46.5 (2)	Pb1—C1—C6—C5	−178.8 (2)
Br2—Pb1—C1—C6	−140.4 (2)	C11—N1—C7—C8	−2.8 (5)
Br2^i^—Pb1—C1—C6	39.6 (2)	N1—C7—C8—C9	−0.8 (5)
Br1—Pb1—C1—C2	−45.5 (2)	C13—N2—C9—C8	−176.7 (3)
Cl1^i^—Pb1—C1—C2	134.5 (2)	C12—N2—C9—C8	−4.9 (5)
Br1^i^—Pb1—C1—C2	134.5 (2)	C13—N2—C9—C10	4.3 (5)
Br2—Pb1—C1—C2	40.6 (2)	C12—N2—C9—C10	176.2 (3)
Br2^i^—Pb1—C1—C2	−139.4 (2)	C7—C8—C9—N2	−174.7 (3)
C6—C1—C2—C3	0.0 (5)	C7—C8—C9—C10	4.3 (5)
Pb1—C1—C2—C3	179.0 (2)	N2—C9—C10—C11	174.6 (3)
C1—C2—C3—C4	−0.3 (5)	C8—C9—C10—C11	−4.4 (5)
C2—C3—C4—C5	0.3 (5)	C7—N1—C11—C10	2.6 (5)
C3—C4—C5—C6	−0.1 (5)	C9—C10—C11—N1	1.1 (5)

Symmetry codes: (i) −*x*+1, −*y*+1, −*z*+1.

### Hydrogen-bond geometry (Å, °)

**Table:**

*D*—H···*A*	*D*—H	H···*A*	*D*···*A*	*D*—H···*A*
N1—H1···Br1	0.87 (1)	2.49 (2)	3.260 (3)	148 (4)
